# Cross-modal neuroplasticity in partial hearing loss: a mini-review

**DOI:** 10.3389/fnins.2025.1627888

**Published:** 2025-11-14

**Authors:** Patricia V. Aguiar, Jennifer Preman, Brandon T. Paul

**Affiliations:** Department of Psychology, Toronto Metropolitan University, Toronto, ON, Canada

**Keywords:** cross-modal plasticity, hearing loss, neuroimaging, visual perception, EEG, fMRI, fNIRS

## Abstract

Sensory loss induces adaptive neural changes in the remaining non-deprived senses, known as cross-modal plasticity. Recent proposals of cross-modal plasticity suggest that it is a top-down, dynamic phenomenon that can occur through the lifespan and is initiated whether sensory loss is total (as in blindness or deafness) or more subtle (mild-to-moderate partial sensory loss). However, it is unclear whether adaptive plasticity differs between total and partial loss, because there is less research on the latter condition. Here we reviewed neuroimaging research on cross-modal plasticity in adult humans with mild-to-moderate hearing loss and compared it to three claims derived from deafness research. First, cross-modal plasticity is thought to involve both intra-modal strengthening of remaining senses and cross-modal recruitment of deprived cortical areas, but we were unable to identify strong evidence of cross-modal recruitment in adults with partial hearing loss. Second, cross-modal plasticity is believed to arise through top-down connections and implicates cognitive function, which agreed with our findings. Third, cross-modal plasticity is believed to enhance perception in the non-deprived senses. No study in our review supported this claim, but it is possible that cross-modal plasticity in partial hearing loss results in stronger modulation of auditory function by intact senses. In addition, many study outcomes in humans with partial loss were inconsistent. Overall, it may be premature to conclude that cross-modal plasticity operates the same for partial and total forms of sensory loss in humans, and we provide several recommendations for testing these claims in future research.

## 1 Introduction

C*ross-modal plasticity* describes when sensory loss, such as blindness or deafness, induces adaptive neuroplastic changes in sensory areas of the cerebral cortex (Bavelier and Neville, 2002). Two forms of cross-modal plasticity across are *intra-modal compensation* and *cross-modal recruitment* (Ewall et al., 2021). Intra-modal compensation is the strengthening of neural processing for the spared senses, for instance, when auditory cortex increases sensitivity to sound stimulation in blindness (Sadato et al., 1996). Cross-modal recruitment is when activity of the spared senses engages brain regions of the deprived senses. For example, visual stimulation activates deaf auditory cortex more than in hearing (Finney et al., 2001), and visual cortex is more strongly activated by sound in blindness (Kujala et al., 1997). Experimental studies demonstrate that cross-modal plasticity causally explains enhanced or “supra-normal” perceptual abilities that develop after sensory loss (Lomber et al., 2010; Meredith et al., 2011) and likely accounts for improved visual motion detection in deafness (Gougoux et al., 2004; Vetter et al., 2020; Voss, 2016). Overall, cross-modal plasticity research shows that the brain adapts to sensory loss by strengthening its existing sensory functions and using available neural resources left by the deprived sense.

Early research on cross-modal recruitment in congenital blindness or deafness led to claims that deprived sensory regions can undergo extensive structural and functional reorganization (Kral, 2007; Makin and Krakauer, 2023). In light of newer evidence, Kral and Sharma (2023) recently proposed that cross-modal plasticity is a more flexible and dynamic phenomenon that does not require dramatic functional rewiring of sensory cortex as was previously believed. Instead, cross-modal recruitment operates by up- and down-regulating preexisting neural connections (Makin and Krakauer, 2023), especially in non-primary regions of sensory cortex that receive modulatory input from external senses. For instance, sensory neurons that are deprived of stimulation can upscale their synaptic inputs via homeostatic plasticity so that they maintain a desired input–output level (Turrigiano, 2008), and synaptic scaling may include inputs from non-deprived senses. Sensory regions are thought to maintain their functional roles because there is no extensive reorganization, but these regions can switch their drive from a deprived sense to a remaining sense because of upregulation. Further, these inputs from non-deprived senses to sensory-deprived regions originate from higher-level association areas implicated in multimodal object perception and cognition (Ghazanfar and Schroeder, 2006), implying that cross-modal plasticity is a top-down process and draws from cognitive resources (Kral and Sharma, 2023).

Kral and Sharma’s (2023) proposal also explains how cross-modal plasticity can arise after partial sensory loss, a sensory status between the absence or near-absence of a sense (what is meant herein by deafness and blindness) and typical sensory function. The development of cross-modal plasticity through existing neural connections means that sensory cortex can adjust to continuous changes in sensory experience, even with mild sensory losses in adulthood. This claim was mainly supported by selected studies on visual cross-modal plasticity after mild-to-moderate hearing loss in middle-aged and older adult humans (Campbell and Sharma, 2014; Glick and Sharma, 2020) as well as noise-exposed animals (Schormans et al., 2017). Treatment of hearing loss with hearing aids also appears to reverse cross-modal plasticity (Glick and Sharma, 2020), affirming that cross-modal plasticity is dynamic and bidirectional. Despite their theoretical importance, studies on visual cross-modal plasticity in human adults with partial hearing loss have not been reviewed in full and weighed against claims made by cross-modal frameworks, including Kral and Sharma (2023). This is noteworthy because mild-to-moderate hearing loss is highly common, especially in aging, and is considerably more prevalent than deafness in humans (Haile et al., 2021). Yet, how sensory cortex adapts under with mild hearing loss is not well described. To fill this gap, we conducted a literature review on neuroimaging studies on visual processing in adults with partial hearing loss. Specifically, we aimed to evaluate three claims based on deafness research: (1) Is there clear evidence of cross-modal recruitment separate from intra-modal compensation in partial hearing loss? (2) Are there top-down or cognitive influences on cross-modal neural activity in partial hearing loss? (3) Is cross-modal plasticity in partial hearing loss linked to any measurable behavioral or perceptual benefits?

## 2 Results and discussion

We used search strings visu*, hearing loss, hearing impair*, cross-modal, and *plasticity* in Web of Science (*N* = 387), PubMed (*N* = 155), and PsycNET (*N* = 74), which yielded 616 total entries. We included studies that used visual-only stimulation or measured visual activity in cortical areas alongside a visual task. We excluded studies on multisensory integration [reviewed elsewhere by Brooks et al. (2018)], unless these studies reported neural responses to visual-only stimulation. Further, we excluded studies that did not have a neuroimaging component, because neural evidence was required to evaluate cross-modal plasticity claims [see Choi et al. (2024) for a review of visual behaviors in hearing loss]. Finally, we excluded studies on cochlear implant users because their hearing loss is commonly profound, and cross-modal effects in cochlear implant users with residual hearing function are not often distinguished from cases of total deafness. Duplicates and entries that did not match the above criteria were removed from search results, resulting in 15 studies for review shown in Table 1.

**Table 1:** Studies measuring visual cortical activity in partial hearing loss.

Study	Participants [Group (*N* = X); age range, mean ± SD (years)]	Participant pure-tone thresholds [*frequency range*. Group: PTA range; mean ± SD in dB HL]^a^	Task	Neuroimaging [modality; ROI; neural measure]	Key result(s) related to HL
Aguiar et al. (2025)	HL (*N* = 67); 53–80, 70 ± 5.5	*2000–8,000 Hz*; HL: 9–76.5; 36.8 ± 16.45	View apparent motion (circle-star pattern, lips closed to open “oh” articulation).	EEG; parietal, occipital; VEP; oscillations	Greater HL was associated with delayed N1 latency for lip stimuli, earlier P2 latency, smaller P2 amplitudes, increased oscillatory power for circle-star stimuli, and greater auditory cortical activation.
Campbell and Sharma (2014)	HL (*N* = 9); 38.4–68.2, 56.9 ± 28.9	*250–1,000 Hz*; HL: <25; TH: <25	View apparent motion (circle-star pattern).	EEG; occipital; VEP	The HL group showed larger P1, N1, P2 amplitudes and shorter N1 latency. N1 latency decreased with greater HL and worse speech-in-noise perception.
	TH (*N* = 8); 37.4–57, 50.5 ± 26.2	*2000–8,000 Hz*; HL: Mild-to-mod HL; TH: <25			
Campbell and Sharma (2020)	Mild HL (*N* = 10); 38.4–69.6, 58.6 ± 9.5	*500–2000 Hz*; Mild HL: <25; Mod HL: <25	View apparent motion (circle-star pattern).	EEG; frontal; VEP	The moderate HL group showed earlier P1 latency in both left and right frontal cortices and reduced P2 amplitude in the left frontal cortex. P1 latency in the left frontal cortex decreased with worse speech-in-noise perception. P2 amplitude in left- and right-frontal cortices decreased with greater HL.
	Mod HL (*N* = 7); 54.5–78, 66 ± 7	*4,000–8,000 Hz*; Mild HL: 25–36; Mod HL: 45–52			
Glick and Sharma (2020)	HL (*N* = 21); n.r., 65.4 ± 4.23	*500–2000 Hz*; HL: 27.08 ± 10.41; TH: <26; 10.58 ± 5.23	Longitudinal: baseline and 6-month post-bilateral hearing aid fitting in HL group.	EEG; frontal, pre-frontal, occipital, temporal; VEP	At baseline, the HL group showed earlier right temporal P1, N1, and P2 latencies compared to the TH group. Post-treatment, these latencies were later in the HL group, with no significant group differences.
	TH (*N* = 13); n.r., 62.62 ± 4.91	*2000–6,000 Hz*; HL: 47.44 ± 11.54; TH: <26; 13.91 ± 3.77	View apparent motion (circle-star pattern).		Within the HL group at baseline, greater high-frequency HL and worse speech-in-noise perception were correlated with earlier right temporal P1, N1, and P2 latencies.
Liang et al. (2020)	Mod-to-severe HL (*N* = 10); 26–52, n.r. ± n.r.	*250–4,000 Hz*; Mod-to-severe HL: average hearing thresholds from 60–80	View sound (e.g., playing instrument) and non-sound (e.g., reading book) images.	EEG; frontal-temporal, occipital; VEP	HL group had larger P1 and N2 amplitudes in frontocentral channels. HL group had shorter N2 latencies for non-sound images.
	TH (*N* = 10); “age-matched”	TH: “normal hearing”			
Loughrey et al. (2023)	Mild HL (*N* = 21); n.r., 70.6 ± 3.9	*500–6,000 Hz (Better ear)*; Mild HL: 32.9 ± 3.6	Working memory task; view three shapes (shapes and colored shapes conditions), then indicate if new or same after brief interval.	EEG; frontocentral, centro-parietal, parieto-occipital; VEP	No behavioral differences between groups. Several differences between groups depending on task condition and sensor location. Generally, HL associated with larger P1 and P2 amplitudes, and smaller P3 and late positive potentials. No correlation between cortical activation and task performance.
	Mod HL (*N* = 23); n.r., 73.5 ± 6.4	Mod HL: 50.9 ± 11.8			
	TH (*N* = 14); n.r., 69.1 ± 3.3	TH: 20.3 ± 2.9			
Mai et al. (2024)	Adults (*N* = 10); 63–78, 70 ± 4.5	*250–6,000 Hz*; *N* = 2: <24	Longitudinal: Baseline (T0), following 4 weeks SIN training (T1), following 4 weeks training cessation (T2).	fNIRS; auditory; visual-evoked activation and functional connectivity	No change in auditory cortical activation with training. Connectivity between auditory and higher-level non-auditory regions (parietal, frontal, temporo-parietal) decreased post-training (T1), returning to baseline at follow-up (T2).
		*2000–8,000 Hz*; *N* = 8: 30–60	View reversing radial checkerboard.		
Paul et al. (2025)	Adults (*N* = 25), 18–78, 41 ± 19.7	*4,000–8,000 Hz*; Adults: −1.25–55; 17.90 ± 16.29	Working memory task; indicate if target word was part of 5-word sentences (shown word-by-word).	EEG; parietal, occipital; VEP	Greater HL was associated with increased N1 amplitude in right auditory cortex and delayed N1 and P2 latencies.
Puschmann and Thiel (2017)	HL (*N* = 20); 52–67, 61 ± 5	*2000–8,000 Hz*; HL: Normal-to-severe HL	View dot motion stimuli.	fMRI; Whole-brain BOLD responses (visual task > rest).	No significant relationship between degree of HL and neural activation.
Rosemann and Thiel (2018)	HL (*N* = 20); n.r., 63.5 ± 5.4	*125–1,000 Hz*	Select noun from visual-only sentences in 2AFC task. Select phoneme from visual-only phonemes (McGurk) in 4AFC task	fMRI; Whole-brain BOLD responses (by-group contrasts).	HL associated with more frequent McGurk illusions, but no difference in audiovisual sentence task. Visual sentences led to higher frontal activation with HL, and McGurk illusion frequency was positively correlated with middle, medial, and inferior frontal gyrus activation. No evidence of cross-modal activation.
	TH (*N* = 19); n.r., 63.2 ± 5	HL: 6.14 ± 6.93;TH: 6.52 ± 5.16; *2000–8,000 Hz*; HL: 39.52 ± 10.25; TH: 18.92 ± 5.63			
Rosemann and Thiel (2020)	HL (*N* = 19); n.r., 64.6 ± 6.3	*2000–8,000 Hz*	N-back working memory task with face and house images.	fMRI; Whole-brain BOLD responses (by-group contrasts).	No differences in working memory task performance and no differences in neural activation.
	TH (*N* = 19); n.r., 63.3 ± 5.4	HL: 41.4 ± 9.14; TH: 19.9 ± 6.76			
Seol et al. (2024)	HL (*N* = 4); 54–61, 60.0 ± 4.1	*500–4,000 Hz (Right/Left averages)*	View reversing radial checkerboard.	EEG; occipital; VEP	The HL group and cochlear implant group showed descriptively smaller P1 amplitudes, compared to the TH group, but no statistical analysis was reported.
	TH (*N* = 7); 23–44, 31.6 ± 8.8	HL: 62.5/59.7; TH: 7.1/5.9			
Shende et al. (2024)	HL (*N* = 19); n.r., 71.53 ± 8.04	500–4,000 Hz (better ear)	Encode and recall visual word lists; words assigned high and low value “points” for recall.	EEG; frontal; oscillations	Increased theta synchronization 100–200 ms after stimulus onset in HL group. HL scored lower on visual word recall.
	TH (*N* = 17); n.r., 67.76 ± 4.92	HL: 32.63 ± 7.05; TH: 17.13 ± 5.43			
Stropahl and Debener (2017)	Mild-to-moderate HL (*N* = 18); n.r., 69.3 ± 1.68	*1,000–4,000 Hz*	View 1 s static image of neutral face.	EEG; auditory; VEP	No significant auditory cortex activation in mild-to-moderate HL or cochlear implant groups. Descriptively higher cross-modal activation in the mild-to-moderate HL group compared to the TH group, but not significant.
	Cochlear-implant (*N* = 18); n.r., 58.5 ± 3.8	Mild-to-mod HL: 24–60; 42 ± 3			
	TH (*N* = 17); n.r., 57.2 ± 4.3	TH: <35			
Zhu et al. (2024)	HL (*N* = 30); 60–73, 66.6 ± 3.8	*500–4,000 Hz*; HL: 45.2 ± 7.6	Detect target circle (left/right visual field) from a circular array of 12 shapes (11 diamonds).	EEG; parietal-occipital; VEP, contralateral-minus-ipsilateral difference waves	No significant differences in task accuracy across groups, or in reaction time across older adult groups. HL did not predict task accuracy or reaction time, but worse speech-in-noise perception was correlated with worse task performance (effects were not seen for reaction time).
	TH older adults (*N* = 30); 60–70, 65.4 ± 3.3	TH older adults: 15.7 ± 4.4			The HL group showed decreased N2pc amplitude compared to TH younger and older adult groups. N2pc amplitude decreased with greater HL and worse speech-in-noise perception.
	TH young adults (*N* = 35); 22–29, 24.5 ± 2.3	TH young adults: 6.3 ± 3.1			

TH, typical hearing; HL, hearing loss; dB HL, decibels hearing level; mod, moderate; PTA, Pure Tone Average; EEG, electroencephalogram; fMRI, functional magnetic resonance imaging; fNIRS, functional near infrared spectroscopy; VEP, visual evoked potential; ROI, region of interest; avg., average; n.r., not reported; Hz, Hertz.

a All available values are reported. Missing data are left blank rather than labeled as “n.r.”; in these cases, maximum cut-off values were included where possible. Italicized values represent the frequency range over which audiometric thresholds were collected.

### 2.1 General summary

Thirteen of the reviewed studies in Table 1 included middle-aged to older adults with moderate or mild-to-moderate hearing loss. Liang et al. (2020) recruited moderate-to-severe hearing loss in younger to middle-aged adults, and Paul et al. (2025), recruited younger and older adults with only high-frequency hearing loss. Eight studies examined neural activity during passive visual stimulation, and seven included active behavioral tasks. Eleven studies used electroencephalography (EEG) to measure visual evoked potentials or neural oscillations. Four studies measured hemodynamic responses and functional connectivity using functional magnetic resonance imaging (fMRI) or functional near-infrared spectroscopy (fNIRS).

The visual evoked potential (VEP) was the most common technique used to measure cross-modal plasticity in humans with partial hearing loss. The VEP complex consists of ordered components labeled P1, N1, P2, N2, etc., that represent the maxima and minima of phase-locked voltage fluctuations along the visual cortical processing pathway. VEPs are characterized by their amplitude and latency. When measured by scalp EEG, VEP components reflect a composite of multiple underlying cortical generators that sum at the surface sensors (Arroyo et al., 1997; Freunberger et al., 2007). Seven of eleven EEG studies reported correlations between hearing loss and VEPs. Several studies claim that visual cross-modal plasticity occurs when hearing loss coincides with larger VEP amplitudes and shorter VEP latencies, which purportedly indicate more sensitive or efficient visual processing. Shown in Table 2, this configuration of results was observed in 16 cases (i.e., results of an analysis). However, study outcomes were highly inconsistent. The opposite effect, where smaller amplitudes or longer latencies occurred with increasing hearing loss, were reported in 8 instances, and 24 sets of analysis across VEP components were not significant. Effects on the P2 component were the least consistent in direction and spatial location, while effects on the P1 and amplitude of N1 were more consistent.

**Table 2:** Statistically significant effects of hearing loss on visual cortical potentials in EEG studies.

Study	Visual stimulus	P1 amplitude	P1 latency	N1 amplitude	N1 latency	P2 amplitude	P2 latency	N2 amplitude	N2 latency	Other
Aguiar et al. (2025)	Lip motion/apparent motion^a^	n.s.	n.s.	n.s.	**↑ (occ; lip)** n.s. (motion)	**↓ (occ; lip)** n.s. (motion)	**↓ (occ; lip)** n.s. (motion)	–	–	–
Campbell and Sharma (2014)	Apparent motion^a^	**↑ (occ)**	n.s.	**↑ (occ)**	**↓ (occ)**	**↑ (occ)**	n.s.	–	–	–
Campbell and Sharma (2020)	Apparent motion^a^	n.s.	**↓ (front)**	n.s.	n.s.	**↓ (front)**	n.s.	–	–	–
Glick and Sharma (2020)	Apparent motion^a^ (baseline)	n.s.	**↓ (R temp)**	n.s.	**↓ (R temp)**	n.s.	**↓ (R temp)**	–	–	–
Liang et al. (2020)	Sound and non-sound images	**↑ (front/cent)**	n.s.	n.s.	n.s.	–	–	**↑ (front/cent)**	**↓ (front/cent; non-sound)**	–
Loughrey et al. (2023)	Shapes/color (encoding)	**↑ (occ)**	n.s.	**↑ (occ)**	n.s.	**↑ (par, mild HL) ↓ (par, mod HL)**	n.s.	–	–	**↓ (P3) ↓ (Late positive)**
Paul et al. (2025)	Visual text	n.s.	n.s.	**↑ (occ)**	**↑ (occ)**	n.s.	**↑ (occ)**	–	–	–
Seol et al. (2024)	Reversing radial checkerboard	↓ (occ) (not tested)	–	–	–	–	–	–	–	–
Zhu et al. (2024)	Shapes	–	–	–	–	–	–	**↓ (occ)**	–	–

Arrows indicate the direction of the effect (increased or decreased amplitude or latency), and the scalp location is provided in parentheses. n.s., not significant; occ, occipital; front, frontal; temp, temporal; cent, central; par, parietal; HL, hearing loss. Dashed lines indicate that results were not applicable or not reported.

a Stimulus was a concentric gradient that morphed to a radially modulated gradient, giving rise to apparent motion (Doucet et al., 2005). Boldface text indicates effects that were statistically significant.

Multiple factors potentially explain inconsistent correlations between VEP components and hearing loss. VEPs were evoked by several types of stimuli, including visual speech (Aguiar et al., 2025), motion (Aguiar et al., 2025; Campbell and Sharma, 2014, 2020; Glick and Sharma, 2020), written words (Paul et al., 2025) shapes (Loughrey et al., 2023; Zhu et al., 2024), reversing checkerboards (Seol et al., 2024) and photographs (Liang et al., 2020), which could change VEP morphology because they are processing along separate anatomical pathways (Harter et al., 1982). Some studies included an active task, and attention and memory demands are known to modulate VEP amplitudes and latencies (Adam and Collins, 1978; Eason, 1981). Participant-level factors, such as age degree of hearing ability, and cognitive function (Glick and Sharma, 2020) also add to variability to the data and could affect correlations between hearing sensitivity measures and VEP features. It is also possible that the association between hearing loss and neural activity indexed by VEPs is not sufficiently robust for evaluating claims about cross-modal plasticity. Beyond VEP analysis, both Shende et al. (2024) and Aguiar et al. (2025) analyzed time-frequency representations of EEG data and found increased theta or alpha oscillation synchronization occurring 100–200 ms after the onset of visual stimulation.

Neural activation and functional connectivity recorded with fMRI was less frequently used to measure cross-modal responses. Rosemann and Thiel (2018) found increased activation in frontal areas to visual speech in participants with hearing loss, and frontal activation in medial, middle, and inferior frontal gyrus was correlated with an increased visual bias in an audiovisual speech task. However, there was no evidence for visual cross-modal recruitment of auditory cortex. Two other fMRI studies did not find effects of hearing loss on neural activation to visual images or visual motion (Puschmann and Thiel, 2017; Rosemann and Thiel, 2020).

All but two studies reviewed in Table 1 used cross-sectional and correlational designs. The longitudinal study conducted by Glick and Sharma (2020) measured VEPs in older adults with age-related hearing loss before and after 6 months of hearing aid use. VEP P1, N1, and P2 latencies over right temporal cortex were shorter in the hearing loss group compared to a control group prior to provision of hearing aids, but latencies returned to levels comparable to typical hearing adults following the intervention. This suggests that visual cross-modal plasticity can be reversed by hearing treatment. In a complementary study, Mai et al. (2024) used fNIRS to measure hemodynamic responses to visual checkerboard stimulation in people with hearing loss, before and after 4 weeks of speech-in-noise training. There were no effects on auditory cortical activation, but training decreased functional connectivity between auditory, frontal, parietal and temporo-parietal areas, with this effect disappearing after 4 weeks. The decrease in frontotemporal connectivity suggested reduced visual cross-modal recruitment because of increased auditory experience.

### 2.2 Claim 1: cross-modal recruitment in partial hearing loss

We first examined evidence for visual cross-modal recruitment of auditory cortex in partial hearing loss. Research in animal models shows that visual recruitment occurs in higher-order, non-primary auditory cortex (Lomber et al., 2010; Meredith et al., 2011). Here, neurons are innervated by pre-existing “heteromodal” visual or somatosensory synaptic inputs that typically modulate auditory processing in an inhibitory fashion and affect the phase of ongoing neuronal oscillations (Kayser et al., 2008; Morrill and Hasenstaub, 2018). Kral and Sharma (2023) propose that deafness reduces input to higher-order cortical neurons, and homeostatic mechanisms cause heteromodal inputs to strengthen and transition from a modulatory to a driving role. This raises visual activity levels in higher-order auditory regions and results in cross-modal recruitment. Neural activity recorded from a rat model of noise-induced hearing loss also suggests that cross-modal recruitment can occur with partial hearing decline. Noise-exposed animals show an increase in the number of neurons responding to visual and audiovisual stimulation in higher-order auditory and multisensory cortex compared to typical hearing control rats (Schormans et al., 2017; Schormans and Allman, 2024). These data also show evidence for a transitional shift in the functional border between auditory and visual areas across sensory cortex, where auditory-responding regions recede and visual-responding regions expand. The expansion of visual-responding areas across sensory cortex with cross-modal recruitment might be expected to arise in human neuroimaging research as increased activity in auditory temporal regions anterior to visual cortex.

VEPs measured by scalp EEG cannot definitively resolve neural sources because volume conduction through skin and other tissues distorts the distribution of neuroelectric potentials. Underlying neural generators can only be approximated by using spatial filtering and modeling of tissue conductance. Of seven EEG studies that employed source analysis, two found that hearing loss was associated with increased right-sided or bilateral temporal cortex activation, consistent with evidence of cross-mod recruitment (Aguiar et al., 2025; Paul et al., 2025). Four additional studies using source analysis to resolve VEP generators found descriptive evidence for activation of temporal cortical areas (Campbell and Sharma, 2014, 2020; Glick and Sharma, 2020; Liang et al., 2020), but these studies did not statistically compare these activations across hearing loss and typical hearing groups. One additional study did not find statistically significant differences in auditory cortical source activation between hearing loss and typical hearing groups (Stropahl and Debener, 2017). Notably, no fMRI or fNIRS study in this review showed evidence for cross-modal recruitment. It is possible that visual cross-modal recruitment does not alter metabolic demands in auditory or multi-sensory regions, but this claim cannot be inferred from null results.

Taken together, only a few EEG studies showed statistical evidence for cross-modal recruitment in adult humans with partial hearing loss, while others only showed qualitative differences in activation. Note that source analysis in EEG studies does not rule out the possibility for “leakage” of intra-modal activity into the source activity. If the observed changes in VEP components with hearing loss result from cross-modal plasticity, then it is likely that intra-modal plasticity plays a substantial role. Resting-state fMRI analysis have shown increased resting-state functional connectivity in visual subnetworks in groups with hearing loss (Ponticorvo et al., 2022) and increased connectivity between visual and cognitive areas (Lian et al., 2025). These studies were not included in our main review because no visual stimulation was used, but they suggest that modulation of VEPs by hearing loss can arise through intra-modal compensation.

### 2.3 Claim 2: top-down influence on cross-modal plasticity

The heteromodal inputs into non-primary auditory cortex through which visual cross-modal recruitment is proposed to develop are top-down modulatory connections originating high-level multimodal regions implicated in cognition (Ghazanfar and Schroeder, 2006). With sensory deprivation, these top-down hetero-modal connections are proposed to upregulate through homeostatic compensatory plasticity and become the driving input, allowing these regions to perform their functional roles (Kral and Sharma, 2023). Engagement of cognitive neuromodulatory systems such as the basal forebrain cholinergic system are top-down and experience-dependent and have also been shown to gate cortical sensory plasticity after sensory loss (Ramanathan et al., 2009). In humans with deafness, visual cognitive tasks activate temporal cortex, providing evidence for top-down involvement in cross-modal plasticity (Cardin et al., 2018). In partial hearing loss, recruitment of frontal brain regions has been observed during auditory speech tasks and is hypothesized to reflect a compensatory increase in resources for cognitive functions such as effort (Pichora-Fuller et al., 2016) or working memory (Rönnberg et al., 2010). Cross-modal plasticity may also draw from compensatory cognitive resources.

Three studies in Table 1 showed evidence for frontal activation when visual stimulation was used. One used fMRI to show increased frontal activation when participants viewed visual sentences (Rosemann and Thiel, 2018), and two showed descriptively higher frontal activation sourced from VEPs evoked by a motion stimulus (Campbell and Sharma, 2020; Glick and Sharma, 2020). Another study did not find increased frontal activity in hearing loss when using a visual moving dot stimulus but did find higher resting state connectivity between visual and frontal regions (Puschmann and Thiel, 2017). In 10 adults with partial hearing loss, Mai et al. (2024) used fNIRS to show that speech-in-noise training reduced frontotemporal connectivity during visual stimulation. This suggested that increased auditory activity levels attenuated cross-modal activity patterns. One final fMRI study that presented faces and images in a working memory task found no differences in frontal activation between hearing loss and control groups (Rosemann and Thiel, 2020). Therefore, with some exception, these studies agree with claims that cross-modal plasticity arises through top-down connections and implicates frontal cognitive brain networks.

Some studies in Table 1 were not conducted to test for cross-modal plasticity and were instead designed to identify evidence of cognitive decline. Hearing loss has been reliably shown to increase the risk of dementia and cognitive impairment (Deal et al., 2017; Lin et al., 2011, 2023) although no causal relationship has been established. One hypothesis suggests that hearing loss increases the demand on cognitive resources, which may include strategies to increase reliance on visual perception. However, long-term demands of cognitive resource use may degrade cognitive flexibility and function, resulting in or exacerbating cognitive impairment and dementia (Slade et al., 2020). Some EEG studies in Table 1 used visual stimulation in attention and memory tasks to measure cognitive ability, because auditory stimulation would be affected by hearing loss. Zhu et al. (2024) found worse visual attention performance correlated with smaller cortical N2 responses in older adults with partial hearing loss, consistent with cognitive decline. Loughrey et al. (2023) reported smaller amplitudes for the P3 and late positive potential in a group of adults with hearing loss, but no difference was found for performance on a working memory task. Loughrey et al. (2023) also found that the earlier cortical potentials P1 and N1 were higher in amplitude in the hearing loss group, which was interpreted as cross-modal plasticity. These data may suggest that cross-modal plasticity can occur with cognitive-decline, but the absence of behavioral differences casts doubt on this interpretation.

Clarifying evidence was provided by Glick and Sharma (2020) who conducted a longitudinal study to show that several months of hearing aid use reversed EEG evidence of cross-modal plasticity. The authors also tracked how cognitive function changed before and after the intervention. At baseline, VEP evidence of cross-modal plasticity in participants with hearing loss was correlated with worse performance on multiple cognitive tasks including processing speed and working memory. After 6 months of hearing aid use and the reversal cross-modal plasticity, performance on the cognitive tasks improved. One interpretation is that cross-modal plasticity exhausted cognitive reserves and lowered cognitive performance, which could be alleviated with hearing restoration. However, it is also possible that cognitive effects and cross-modal VEP plasticity are independent and were indirectly related through their correlation to hearing loss.

Both cognitive decline and partial hearing loss are more common in older age, but little is known about age effects on cross-modal plasticity. Studies in Table 1 often include older adults, but do not commonly attempt to model age-related effects separately from hearing loss effects. EEG studies show that VEP latencies increase with age, especially for the P1 response (Alain et al., 2022; Price et al., 2017; Shaw and Cant, 1980), but VEP latencies are expected to decrease with visual cross-modal plasticity due to increased visual efficiency (Campbell and Sharma, 2014). To address this, both Aguiar et al. (2025) and Paul et al. (2025) examined effects of participant age, respectively, using visual speech or visual text. Interestingly, both studies found statistically independent and inverse effects of age and hearing loss on the visual P2 response. Aguiar et al. (2025) found shorter latencies with hearing loss and longer latencies with age, whereas Paul et al. (2025) found the opposite: shorter latencies with age, and longer latencies with hearing loss. As mentioned, stimulus differences and task requirements could account for the inconsistent direction of these correlations, but these studies nonetheless show evidence for diverging effects of age and cross-modal plasticity on visual processing.

### 2.4 Claim 3: cross-modal plasticity and changes to visual behaviors

Cross-modal recruitment is believed to be responsible for enhanced perceptual abilities found in deaf or blind populations. Improvement in perceptual ability is thought to be constrained to functions that are shared between senses. This is because cross-modal recruitment develops through pre-existing neural connections, allowing sensory cortical areas retain their functional role when switching their response preference from a deprived sense to a remaining sense, which overall supports “supra-modal” perception of objects or events (Kral and Sharma, 2023). Deprived regions do not take on new functions that are specific to remaining senses. In agreement, visual “unimodal” functions, such as color and contrast perception and foveal acuity, and contrast, do not appear to improve with deafness [reviewed in Alencar et al. (2018)] as there is no auditory analogue. On the other hand, visual object motion detection and peripheral object tracking, which are both supported by auditory and visual systems, are enhanced in deaf humans (Codina et al., 2011; Shiell et al., 2014).

Causal evidence for a relationship between cross-modal plasticity and enhanced visual ability comes from the congenitally deaf cat. In the deaf cat auditory cortex, Lomber et al. (2010) found cross-modal recruitment in higher-order areas including the posterior auditory field (PAF) and dorsal zone (DZ). In hearing cats, these areas are, respectively, implicated in auditory localization and auditory motion detection. Congenitally deaf cats also show evidence for enhanced visual localization and visual motion detection. Lomber et al. (2010) used cooling loops to temporarily deactivate PAF and DZ. PAF deactivation abolished deaf cats’ visual localization advantage but not visual motion detection, and DZ deactivation abolished visual motion detection but not visual localization. This double dissociation provides strong causal evidence that cross-modal plasticity supports enhanced visual ability. Auditory cortical areas maintained their “supra-modal” role (object localization in PAF, and object motion in DZ) but utilized visual information delivered through heteromodal inputs to PAF and DZ to perform their function. A follow-up study in congenitally deaf cats confirmed this finding, showing that the auditory field of the anterior ectosylvian sulcus (FAES), a region normally involved in acoustic orienting in hearing cats, is visually reorganized in deaf cats. Temporary FAES deactivation using cooling loops caused visual orienting deficits in deaf cats, again showing a retention of function in cross-modally affected regions (Meredith et al., 2011).

However, no studies in our review showed enhanced visual function or a relationship between visual neural activity and behavioral performance on perceptual or cognitive tasks in partial adult-onset hearing loss (Table 1). In fact, Zhu et al. (2024) found *worse* visual attention in participants with poorer auditory function, which was interpreted as evidence of cognitive decline. While our review cannot corroborate the claim that cross-modal plasticity affects visual-only abilities in partial hearing loss, behavioral research shows evidence for stronger visual influences during audiovisual perception. Compared to people with typical hearing, those with hearing loss are more affected by visual distractors than auditory distractors (Puschmann et al., 2014), are more likely to experience illusory McGurk fusions of auditory and visual phonemes (Rosemann and Thiel, 2018; Stropahl and Debener, 2017) and show larger visual enhancements of neural speech tracking (Puschmann et al., 2019). In the brain, increased visual activation correlates to the susceptibility of experiencing McGurk illusions (Rosemann and Thiel, 2018), and partial hearing loss is associated with increased connectivity between auditory and visual areas during audiovisual stimulation (Puschmann and Thiel, 2017).

Together, these studies suggest that cross-modal plasticity in partial hearing loss is more likely to result in altered visual modulation of auditory function, rather than altered visual function *per se.* At the neural level, visual heteromodal inputs to higher-order auditory cortical neurons may increase in strength but remain modulatory (or a mixture of modulatory and driving) in partial hearing loss because of residual auditory activity. When auditory deprivation rises to deafness, the absence of auditory input permits heteromodal inputs to fully drive auditory cortical neurons (Kral and Sharma, 2023). Visual modulation of auditory function may also occur in multisensory cortex in partial hearing loss, as shown in animal models (Schormans et al., 2017; Schormans and Allman, 2024).

It does not appear that cross-modal plasticity in partial hearing loss is associated with *enhanced* audiovisual abilities as is found in deafness. For example, some studies show reduced or no change in audiovisual perception in mild-to-moderate hearing loss compared to typical-hearing controls (Gieseler et al., 2018; Tye-Murray et al., 2007). In addition, research showing more frequently experienced McGurk illusion in partial hearing loss (Rosemann and Thiel, 2018; Stropahl and Debener, 2017) does not mean the visual influence is beneficial for accurate perception, as is sometimes inferred. Visual events are in fact more distracting (Puschmann et al., 2014). In rats, noise-induced hearing loss impaired perception of timing for audiovisual events (Schormans and Allman, 2019). Therefore, the putative increase in visual modulation of auditory perception with partial hearing loss is not beneficial *per se.* It is also possible that cross-modal plasticity benefits perceptual ability only if these abilities strengthen through experience. In other words, the visual benefits consequent of cross-modal plasticity in partial hearing loss are not spontaneous, exposure and training are needed to improve target functions. For example, adults with mild-to-moderate hearing loss who purposefully rely on visual information as a coping strategy may accrue a visual benefit over time through cross-modal plasticity, but those without long-term experience will not.

## 3 Conclusions and future directions

The objective of this review was to determine whether cross-modal plasticity in partial hearing loss was consistent with its manifestation described in deafness research. At a general level, we found that hearing loss was associated with altered visual neural activity as predicted with cross-modal plasticity, but the direction and specificity of effects was inconsistent. The first of three claims we surveyed was if cross-modal recruitment was evident in partial hearing loss. We found only few studies in humans observed recruitment effects and these findings were derived from EEG techniques with less robust spatial resolution. Second, the reviewed studies agreed with claims that cross-modal plasticity develops through top-down connections and implicates cognitive function. Third, there was no clear visual perceptual benefit linked to cross-modal plasticity in partial hearing loss as is found in deafness, although it is plausible that plasticity with partial loss is associated with stronger visual modulation of auditory function. No study that was reviewed established that cross-modal plasticity was categorically different between partial and total forms of sensory loss, but major claims such as recruitment and modified perceptual ability were not fully corroborated. In addition, study findings in partial hearing loss were unstable or were not often replicated. Overall, there is evidence that visual cortical activity is modulated by hearing sensitivity and experience in humans, but without additional research it may be premature to conclude that it reflects cross-modal plasticity mechanisms known to occur in total sensory loss.

Future research can make several improvements to better test claims that the development of cross-modal plasticity relies on the same mechanisms in both total and partial sensory loss. First, research needs to establish reliable markers of cross-modal plasticity in human neuroimaging studies. For example, effects of hearing loss on VEPs were often identified but were not consistent across studies, even when the same stimulus was used. Multiple cross-modal plasticity studies in partial hearing loss and deafness use a stimulus where an image of a concentric circles in a contrast gradient is followed by another image where the circles are radially modulated into a star (Aguiar et al., 2025; Campbell and Sharma, 2014, 2016, 2020; Doucet et al., 2005, 2006; Fullerton et al., 2023; Glick and Sharma, 2020). The alternation of images is designed to give rise to the impression of apparent motion, a circle morphing into a star, etc., and motion perception has been shown to improve with cross-modal plasticity in deafness (Lomber et al., 2010). However, amplitude, latency, and scalp region effects across different data sets were inconsistent when this stimulus was used (Table 2). Cross-modal plasticity may also be stimulus-specific and sensitive to top-down demands, which creates challenges for developing a robust measure. A neural marker of cross-modal plasticity must have strong test–retest stability and also show the degree of cross-modal recruitment separately from intra-modal compensation Finally, a neural marker would ideally correspond to a measurable change in behavior, so that it is possible to falsify the claim that cross-modal plasticity drives perceptual improvement in cases of partial hearing loss.

A second recommendation is to increase use of longitudinal research designs. Only two studies used longitudinal or training designs in our review, and both suggested that altered visual cortical activity can diminish with hearing restoration or through auditory experience (Glick and Sharma, 2020; Mai et al., 2024). Little is known about how cross-modal plasticity evolves over the lifespan or with visual, not auditory, training. Addressing this gap would make it possible to reveal constraints on claims that cross-modal plasticity is flexible and dynamic. With respect to possible constraints, a third recommendation is to better understand how age and cognitive decline relate to cross-modal plasticity. Increased frontal recruitment accompanying visual cortical activity implies that it could draw from cognitive resources and contribute to cognitive decline. Do early stages of cognitive decline impair the development of cross-modal plasticity? Also, some of the reviewed studies suggest the development of cross-modal plasticity could be limited in older age. Understanding the limitations of cross-modal adaptation is essential for predicting potential perceptual benefits in groups with vulnerabilities.

A better understanding of cross-modal plasticity would prove useful in clinical settings. VEPs can potentially be used as an objective neural measure to track the efficacy of hearing aid use on sensory ability and cognitive function as a complement to behavioral testing (Glick and Sharma, 2020). Furthermore, training on lip reading is hypothesized to improve audiovisual speech perception, but the outcome of training for speech function in partial hearing loss is mixed (Bernstein et al., 2022; Schmitt et al., 2023). A better understanding of top-down cross-modal influences could help to design tasks or encourage coping strategies that maximize learning and uptake of visual information.
